# Progress in Fevers

**Published:** 1902-04-26

**Authors:** 


### Progress in Fevers.

(*Continued from page* 48.)

**Typhoid Fever.**—Leigh Canney[27] describes in detail his scheme of an Army Water Corps. He strongly advocates education of men and officers in all sanitary matters, and especially in the necessity of drinking only pure water. Avoidance of impure water should be regarded as a strict moral obligation. Two per cent. of the strength of the army should be told off as a water corps, the provision of "approved" water being their sole duty. Transport for this should have precedence of everything except one day's ammunition. The corps should march between the scouts and the advancing army. By using a cylindrical copper boiler made up of wedge-shaped sections holding 1 pint each, 50 pints (6¼ gallons) of water could be raised to boiling point in 11 minutes at an expenditure of three-quarters of a pint of petroleum. Cooling could be effected in 6 minutes. Two hundred pounds transport would supply 4 pints daily of pure water for one week to each section of 100 men. Elliot and Washbourne[28] conclude as the result of a study of 262 cases of enteric in South Africa, with a mortality of 13·7 per cent., that inoculation is of little practical value and that the type of the disease is practically the same as that met with in England and America. Cardiac collapse was frequent and also phlebitis. No clinical types could be differentiated according to the probable mode of infection. Rolleston[29] noticed that second attacks occurred following previous attacks in England, India, Egypt, or South Africa itself—a fact also commented on by the last-mentioned observers. In this connection he refers to Durham's theory of relapses, that a typical attack is due to infection with several strains of *bacillus typhosus*, and that if one or more strains is feebly represented insufficient protection against such strains may be developed. C. A. Morris[30] records a case of dry arthritis following typhoid in which the hip joint became disorganised. J. W. Washbourne[31] describes several types of the diseaase. In the *toxic* type the patient rapidly dies without special local symptoms. Marked heart weakness, frequently noticed in South African cases distinguishes the *cardiac* type. In the *abdomina* type there is deep ulceration and sloughing of Peyer's patches. Cases of *prolonged fever* occur in which a relapse overlaps the primary fever, whilst very *mild* types of the disease may only be recognised by the occurrence of a relapse. The prolonged infectivity of the urine and fæces greatly enhances the danger of the spread of the disease, and measures directed to the disinfection of these are essential. Twice in his experience severe hæmaturia has followed the use of urotropin. Typhoid bacilli are also found in collections of pus. Widal and Le Sourd[32] describe a case in which the bacillus was found in the pus of an abdominal abscess, and in pure culture in the "oily" subcutaneous collections which developed at the sites of subcutaneous injections. P. Selby[33] strongly advocates the feeding of enterics on whey instead of milk. Mortality has, in his hands, fallen from 15·5 to 2·7 per cent. since using it. Pulse rate diminishes, and the mouth and tongue remain clean. From ½ to 6 pints in the 24 hours are given, but no limit is placed on quantity. It may be flavoured with tea, coffee, or lemon juice. Whey practically consists of a solution of milk sugar, with a small amount of fat, albumin, and salts. It is not easy to see how the patients can exist with such a small amount of nitrogenous food, but the writer has no doubt about the good results obtained practically. W. W. Golden[34] states that attendance on typhoid fever constitutes a fourth of the medical practice of West Virginia. Milk he still considers the diet for typhoid patients, special daily directions being given as to the times and quantities to be observed. M. Gershel[35] has paid special attention to the Widal reaction in the typhoid fever of children. He finds it of great value as a diagnostic sign, especially in those cases which show a close resemblance to pneumonia, meningitis, or other disease.

[27] Lancet, Nov. 16. [28] Ibid., Nov. 2 and Jan. 18. [29] Brit. Med. Jour., Oct. 5. [30] Lancet, Dec. 7. [31] Clin. Jour., Oct. 30. [32] Lancet, Feb. 15, p. 464. [33] Ibid., Nov. 2. [34] Med. Rec., Nov. 23. [35] Ibid.

